# *Candidatus* Dirofilaria hongkongensis as Causative Agent of Human Ocular Filariosis after Travel to India

**DOI:** 10.3201/eid2308.170423

**Published:** 2017-08

**Authors:** Stefan Winkler, Andreas Pollreisz, Michael Georgopoulos, Zsuzsanna Bagò-Horvath, Herbert Auer, Kelvin Kai-Wang To, Jürgen Krücken, Sven Poppert, Julia Walochnik

**Affiliations:** Medical University of Vienna, Vienna, Austria (S. Winkler, A. Pollreisz, M. Georgopoulos, Z. Bagò-Horvath, H. Auer, J. Walochnik);; University of Hong Kong, Pokfulam, Hong Kong, China (K.K-.W. To);; Freie Universität Berlin, Germany (J. Krücken);; Bernhard Nocht Institute for Tropical Medicine, Hamburg, Germany (S. Poppert);; Regio Klinikum, Wedel, Germany (S. Poppert)

**Keywords:** Candidatus Dirofilaria hongkongensis, Dirofilaria repens, India, Austria, ocular filariosis, zoonoses, eye, worm, parasites, parasitic infections, dirofilariosis

## Abstract

We report a human case of ocular *Dirofilaria* infection in a traveler returning to Austria from India. Analysis of mitochondrial sequences identified the worm as *Candidatus* Dirofilaria hongkongensis, a close relative of *Dirofilaria repens,* which was only recently described in Hong Kong and proposed as a new species.

Dirofilariosis, caused by *Dirofilaria repens* or *D. immitis* nematodes, is a zoonotic filarial infection transmitted through the bite of various mosquitoes. The most frequent manifestations in humans are subcutaneously migrating worms and formation of nodules in various body parts (*1*). Increasing numbers of human *D. repens* infections have been reported from Europe, Africa, and Asia (*2*,*3*). Austria was considered nonendemic, until the first autochthonous case in a human was reported in 2006 (*4*) from the most eastern province, the Burgenland, where *D. repens* nematodes were recently also found for the first time in 2 *Anopheles* mosquito species (*5*). We describe a case of imported ocular dirofilariosis caused by the recently newly proposed species *Candidatus* Dirofilaria hongkongensis (*6*).

The patient, a 38-year-old woman, had recurrent eyelid swelling in both eyes and conjunctival inflammation with watery discharge beginning in June 2011 (Technical Appendix Figure, panel A). She visited numerous physicians and, upon various putative diagnoses (ranging from sicca syndrome to burnout syndrome), she received corresponding therapies, including antibiotics, steroids, and acupuncture. From January 2012 on, the eyelid swellings were accompanied by a creeping sensation and occurred more often. In early August 2012, she sought care at the emergency department of a university eye clinic in Vienna, Austria. She had a moving object in her left eye. Slit lamp examination revealed a white slender worm moving subconjunctivally in the temporal part of the left eye (Technical AppendixFigure, panel B). The conjunctiva was opened under topical anesthesia, and a 13-cm worm (Technical Appendix Figure, panel C) was removed (Video) and morphologically identified as a nongravid female of *D. repens* (Technical Appendix, panel D). Results of serologic testing for filariae were negative before and after extraction of the worm, as were results for testing of EDTA blood for microfilariae. Blood test results, including differential blood counts, were within reference ranges throughout the case history. The patient had returned from a 7-week stay in India, including the areas of Goa, Maharashtra, Delhi, and Uttar Pradesh, 3 months before initial onset of symptoms. Her travel history of the preceding 3 years included 4 more trips to India of several weeks each; a 2-week stay in Israel (October 2010); and a 2-week stay in Dubai, United Arab Emirates (July 2009).

**Video:**
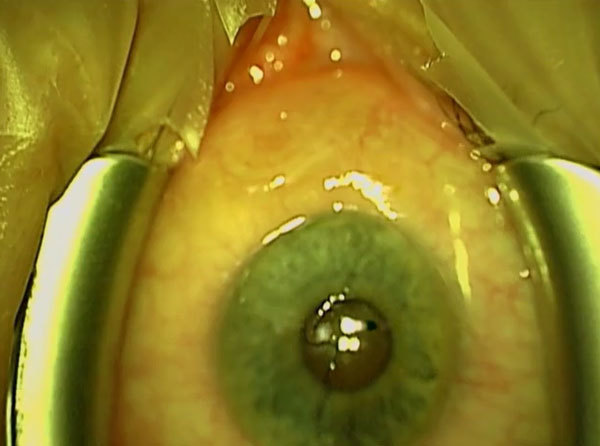
Surgical extraction of a 13-cm worm from the eye of a patient with *Dirofilaria repens* infection (https://www.youtube.com/watch?v=tb9TFpA_YR8).

For confirmation of the morphologic identification, we isolated DNA from a 1-cm piece of the worm after homogenization by using the QIAamp DNA Mini Kit (QIAGEN, Hilden, Germany). We amplified fragments of the cytochrome C oxidase subunit I (COI) with panfilarial primers COXfw 5′-GCKTTTCCTCGTGTTATGC-3′/COXrev 5′-CCAGCCAAAACAGGAACAG-3′ and 12S rRNA with panfilarial primers Panfil-12S-F 5′-gttccagaataatcggcta-3′/Panfil-12S-R 5′-attgacggatgrtttgtacc-3′ (*7*). We sequenced amplicons and subjected them to phylogenetic analyses (online Technical Appendix). All sequence data were submitted to GenBank (accession nos. KY750548–KY750550).

The 329 bp COI fragment (accession no. KY750548) showed 99%–100% identity to 2 sequences from *Candidatus* Dirofilaria hongkongensis (accession nos. KX265050 and JX187591). Identity to *D. repens* sequences was 95%–96%, to *D. immitis* 89%, and to *Onchocerca* spp. up to 92%. The 466 bp mitochondrial 12S rDNA fragment (accession no. KY750549) showed 99% identity to *Candidatus* Dirofilaria hongkongensis sequences from case-patients in India (accession no. KX265050) and Hong Kong (accession no. KY750550), the latter derived from original material of the first description of *Candidatus* Dirofilaria hongkongensis (*6*). Identity to a *Dirofilaria* sp. from a patient returning from India and Sri Lanka and to *Dirofilaria* sp. Thailand II, recently reported among dogs in Thailand (accession nos. KX265092 and KX265093) (*8*), was also 99%. Phylogenetic analysis using the COI sequence clearly placed the sequence into the *Candidatus* Dirofilaria hongkongensis cluster, the sister taxon to *D. repens* (Figure). Although *D. immitis* shows virtually identical COI sequences from 4 continents, genetic variability in *D. repens*–like parasites is obviously much higher, possibly associated with varying zoonotic potentials, reservoirs, and vectors; however, molecular data on *Dirofilaria* are still scarce.

**Figure:**
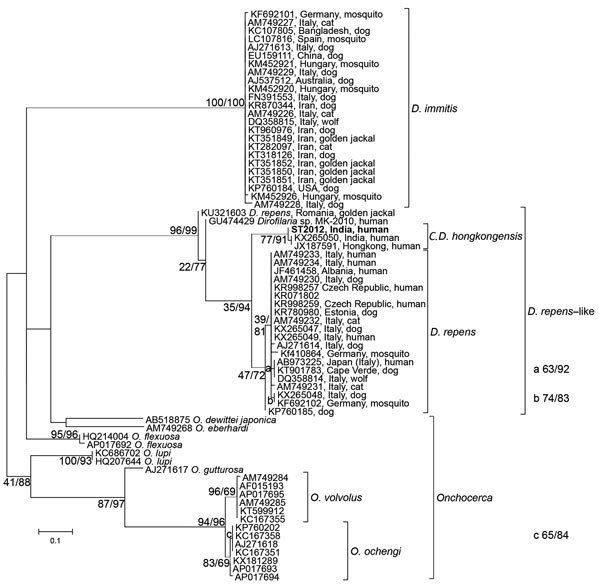
Phylogenetic analysis of the genus *Dirofilaria* based on cytochrome C oxidase subunit I gene sequences from a worm surgically extracted from the eye of a patient who had returned to Austria after travel to India. Bootstrap values and results of the Shimodaira-Hasegawa test are shown before and after the slash. The sequence from the current patient is shown in bold, and clusters within *Candidatus* Dirofilaria hongkongensis, with *Dirofilaria repens* as the sister taxon. Two samples, classified as *Dirofilaria* sp. MK-2010 (GenBank accession no. GU474429) and *D. repens* from Romania (accession no. KU321603), show very high divergence and probably represent different species. The scale bar represents 0.1 substitutions per site. The samples are identified by GenBank accession numbers, country, and host origin, when available. The genera *Dirofilaria* (*D*.) and *Onchocerca* (*O*.) as well as the *Candidatus* status (*C*.) are abbreviated in species names.

In this case, *Candidatus* Dirofilaria hongkongensis was most likely acquired in India. An infection in Austria seems unlikely because, until now, only 1 singular autochthonous *Dirofilaria* infection has been reported, and that case was classic *D. repens* infection (*4*). Dubai is considered nonendemic for *Dirofilaria* spp. parasites, whereas Israel is known to be endemic for *D. repens* nematodes (*1*,*3*), but the patient’s trips to these countries were much longer ago than her latest trip to India. Moreover, all cases from India or Sri Lanka analyzed by us so far represented *Candidatus* Dirofilaria hongkongensis (*8,9*; S. Poppert, unpub. data), suggesting that this species is widely distributed on the Indian subcontinent. In fact, whether classical *D. repens* infection occurs in India at all is unclear. Infections with *Candidatus* Dirofilaria hongkongensis nematodes might take a similar course as infections with classical *D. repens*; however, a case of meningoencephalitis caused by nematodes of this candidate species also has been described (*9*). *Dirofilaria* spp. parasites isolated from human case-patients should be investigated by molecular methods to establish an exact species diagnosis, especially if infections were acquired outside Europe.

Technical Appendix Description of phylogenetic analysis. Images showing the patient’s ocular signs of *Dirofilaria repens* infection and the surgically extracted worm.
