# Supplementary material for: Candidatus Dirofilaria hongkongensis as Causative Agent of Human Ocular Filariosis after Travel to India
Source: Emerg Infect Dis. 2017 Aug;23(8):1428–31. doi: 10.3201/eid2308.170423 (PMC5547781; doi:10.3201/eid2308.170423)
Supplement: Technical Appendix — Description of phylogenetic analysis. Images showing the patient’s ocular signs of Dirofilaria repens infection and the surgically extracted worm. [file 17-0423-Techapp-s1.pdf]

Article DOI: https://doi.org/10.3201/eid2308.170423

Candidatus Dirofilaria hongkongensis as Causative Agent of Human Ocular Filariosis after Travel to India

Technical Appendix

Phylogenetic Analysis

The phylogram includes all cytochrome oxidase sequences for *Dirofilaria*, *Onchocerca volvulus*, and *Onchocerca ochengi* as well as selected sequences for other *Onchocerca* species from GenBank. Sequences were aligned on codon level by using MUSCLE (*1*) as implemented in MEGA6 (*2*). The test for substitution saturation according to Xi et al. (*3*) was negative for both codon positions 1 and 2 and position 3. A phylogenetic model was fitted separately for codon positions 1 and 2 and position 3 by using RAxML (*4*) with the GTR model and 25 Γ distributed rate variation categories among sites and the rapid bootstrap option with 1,000 replicates. The resulting phylogram was also used as input into RAxML to calculate node support with the Shimodaira-Hasegawa modification of the likelihood ratio test.

References

1. Edgar RC. MUSCLE: multiple sequence alignment with high accuracy and high throughput. Nucleic Acids Res. 2004;32:1792–7. PubMed http://dx.doi.org/10.1093/nar/gkh340

2. Tamura K, Stecher G, Peterson D, Filipski A, Kumar S. MEGA6: Molecular Evolutionary Genetics Analysis version 6.0. Mol Biol Evol. 2013;30:2725–9. PubMed http://dx.doi.org/10.1093/molbev/mst197

3. Xia X, Lemey P. Assessing substitution saturation with DAMBE. In: Lemey P, Salemi M, Vandamme A-M, editors. The phylogenetic handbook. Cambridge (UK): Cambridge University Press; 2009. p. 615–30.

4. Stamatakis A. RAxML version 8: a tool for phylogenetic analysis and post-analysis of large phylogenies. Bioinformatics. 2014;30:1312–3. PubMed http://dx.doi.org/10.1093/bioinformatics/btu033

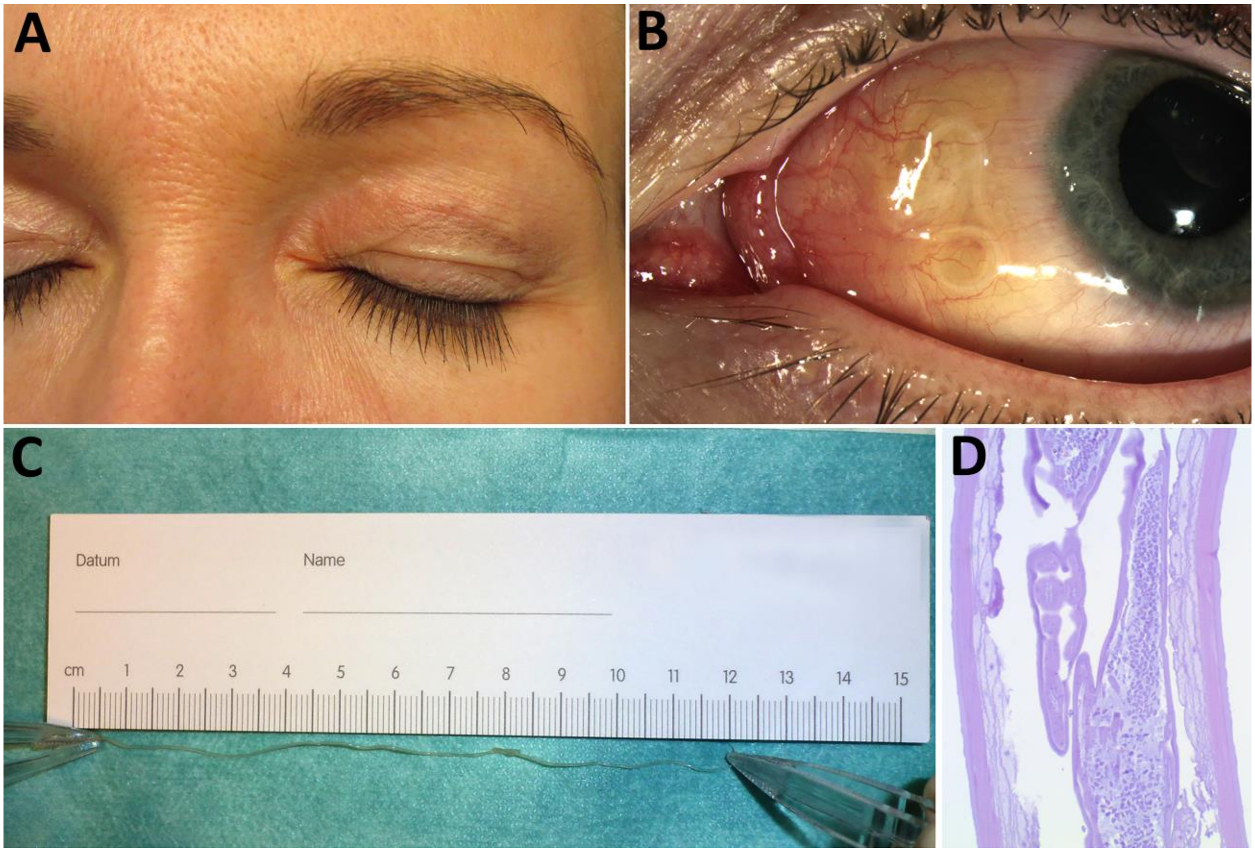

Technical Appendix Figure. Images showing the patient's ocular signs of *Dirofilaria repens* infection and the surgically extracted worm. A) Eyelid swelling (January 2012). B) Worm-like object in the left eye, with anterior chamber and fundus examination revealing no abnormalities (August 2012). C) Size of the extracted worm. D) Longitudinal section of the extracted worm.
